# Phospholipase D inhibition by hexanal is associated with calcium signal transduction events in raspberry

**DOI:** 10.1038/hortres.2017.42

**Published:** 2017-09-13

**Authors:** Walid El Kayal, Gopinadhan Paliyath, J Alan Sullivan, Jayasankar Subramanian

**Affiliations:** 1 Department of Plant Agriculture, University of Guelph-Vineland Station, 4890 Victoria Avenue N, Vineland, Ontario L0R2E0, Canada; 2 Department of Plant Agriculture, University of Guelph, Guelph, Ontario N1G2W1, Canada

## Abstract

Raspberry (Rubus spp.) is an economically important crop with a restricted growing season and very limited fruit shelf-life due to its extreme tenderness. In order to prolong its shelf life, an aqueous composition containing hexanal as the key active ingredient (HC) was applied as a preharvest spray during fruit development. The effects of HC were assessed using physiological, biochemical and anatomical parameters on the treated fruits and compared with the effects of mock inoculation which lacked hexanal. Sugars and acidity did not show a significant change in response to HC treatment, while the pulling force (the tension required to detach the berry from the receptacle) significantly improved in the HC-treated fruits, compared to control. Scanning electron microscope (SEM) analysis revealed a high correlation between the presence of rigid epidermal hairs and a stronger degree of attachment between berries and their receptacle in the HC treated fruits. Further, electron micrographs also showed abnormal crystalline depositions on the epidermal drupelets of the treated berries. Energy Dispersive X-ray Spectroscopy (EDS) analysis showed those crystals to be largely composed of calcium. HC treatment also resulted in the reduction of transcript level of three phospholipase D genes, as well as altered expression pattern of five members of the annexin gene family, and four calmodulin-binding transcription activators. Quantification of PLD activity showed that hexanal inhibited PLD activity in treated berries. The potential crosstalk between hexanal, phospholipase D activity and calcium and this crosstalk’s role in delaying fruit softening and in prolonging storage life of fruits shelf life is discussed.

## Introduction

Raspberry (*Rubus ideaus* L.) is a tender fruit with an extremely short shelf-life. It is a rich source of health-promoting compounds such as anthocyanins. Rapid decline in fruit quality typically occurs due to decay and physiological breakdown, which is associated with the loss of membrane integrity leading to the progression of senescence and stress.^1^ Phospholipase D (PLD), a phospholipid-degrading enzyme is the key enzyme involved in initiating a cascade of catabolic events that leads to the eventual deterioration of the membrane, and is highly active in the fruits of other berry species such as strawberry.^2,3^

Suitable technologies and methods to enhance the postharvest shelf life of tender fruits are in high demand. The application of hexanal as a formulation, either pre- or postharvest, has shown promising results in enhancing the shelf-life of several fruits, vegetables and flowers.^4–6^ Biochemical and genetic studies of fruit softening have indicated that cell-wall processing is the result of coordinated expression of several gene families encoding proteins associated with cell-wall metabolism, including expansins, pectin methylesterases, polygalacturonases, pectate lyases, β-galactosidases, α-L-arabinofuranosidases, endo-(1,4)-β-D-glucanases, β-xylosidases, xyloglucanases, endotransglucosidases, endo-mannanases.^7–11^ However, efforts to suppress expression of cell wall-degrading enzymes by genetic engineering did not provide satisfactory results to efficiently prevent softening of fruits.^12,13^

It has been suggested that inhibition of PLD activity by hexanal application could eventually lead to enhanced membrane stability, and thereby increased longevity of horticultural produce. Previous studies have attempted to correlate increased membrane deterioration that occurs during ripening and senescence to increased phospholipase D activity. Although such an increase in PLD activity was noticeable in some senescing systems such as broccoli florets^14^ and tomato fruit,^11^ it was not as distinct in systems such as carnation flower petals.^15,16^ Thus, increased phospholipid degradation that occurs during ripening/senescence was linked to the activation of PLD by factors such as increase in cytosolic calcium and a decrease in pH, membrane binding promoted by C2 domain, and fatty acid retailoring that increases the availability of preferred PLD substrates.^11,17^ Altering the levels of exogenous calcium application affects parameters for senescence such as protein and chlorophyll content, respiration rates, and cell membrane fluidity.^18^ Other studies also reported that the addition of calcium rigidifies cell wall and obstructs enzymes such as polygalacturonase from reaching active sites.^18^ Calcium plays a special role in maintaining the cell wall structure in fruits and other storage organs by interacting with pectic acid in the cell walls to form calcium pectate. Thus, fruits treated with calcium are generally firmer. The irreplaceable nature of the calcium ion (Ca^2+^) as a signal transduction agent, and in cell wall polysaccharide interactions is undisputed; it is through these processes that calcium is central to stress responses, cell wall structure and remodeling, and to plant tissue development.^19,20^ It has been suggested that calcium rigidifies the cell wall by crosslinking pectic acid residues^21^ and stabilizing cell membranes.^22^ Nevertheless, accumulating evidence indicate that calcium is a universal second messenger, and plays an important role in plant growth and development by mediating response to a variety of environmental and hormonal signals.^23,24^ Signal triggered increase in cytosolic calcium are perceived by calcium sensors. Calmodulin is a ubiquitous calcium sensor, and present in all plant cell types examined thus far.^25,26^ It can modulate the actions of diverse target proteins involved in almost all aspects of cell activity including cell division, cell elongation, ion transport, secondary metabolism, plant defense, etc.^25,26^

Among the calcium-binding proteins, annexins form a family of structurally related proteins that exhibit calcium-dependent binding to phospholipids. Annexins are soluble proteins capable of Ca2+-dependent or independent association with membrane phospholipids.^27^ Genome sequencing revealed that annexins in plants comprise a small multigene family with several members, eight in *Arabidopsis thaliana*^28^ and nine in *Oryza sativa*.^29^ The increased expression of certain annexins uunder specific developmental or environmental challenges has linked them with Ca^2+^ signalling during nodulation,^30^ ABA responses^31,32^ or cold acclimation.^33^ The possibility that annexins form Ca2+ channels, coupled with their presence in root hairs and the root elongation zone, suggests a role in generating the elevated cytoplasmic calcium concentration ([Ca^2+^]cyt) required for tip or extension growth. Plant annexins have been found to have *in vitro* ATPase and GTPase activity.^22,34^ AtANN1 has been identified *in vitro* as an ATP-binding protein.^35^

Our previous study analyzed the effect of hexanal on the relative transcript abundance levels of a set of genes putatively associated with cell wall degrading enzymes in strawberry.^36^ In this study, we report on understanding the mechanism of action of hexanal in delaying membrane deterioration and prolonging shelf life using multidisciplinary approaches including physiological, histological and biochemical characterization. The expression patterns of three phospholipase D genes, five annexin gene members, and four calmodulin-binding transcription activators, were quantified by qRT-PCR during fruit development and ripening in response to hexanal applications. Scanning electron microscopy as well as Energy Dispersive X-ray Spectrometry were conducted to analyze the structural and compositional details of treated and untreated fruits. In addition, phospholipase D activity was assayed during fruit development and six days postharvest after hexanal applications.

## Materials and methods

### Plant material and postharvest treatments

**Figure 1:**
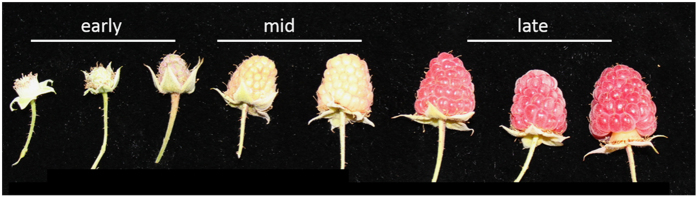
Raspberry fruit development stages.

Fruit were harvested from *Rubus idaeus* (European red raspberry) grown under field conditions (Niagara on the Lake, ON, Canada). For the molecular studies, fruit were selected at three developmental stages (Figure 1): early (small white receptacle with green drupelets); mid (white receptacle with enlarged green drupelets) and late (fully red drupelets). To evaluate the physiological and molecular effects of hexanal on fruit quality and shelf life longevity, fruit were subjected to three pre-harvest sprays with a composition containing hexanal as described in Misran *et al.*^37^

The experiment was carried out in a completely randomized block design with four replications. Each plot was divided in half, one half of the plot was sprayed with hexanal formulation while the other half was sprayed with carrier formula (all ingredients, except hexanal) and used as control. The study was performed for two consecutive growing seasons 2015 and 2016.

### Fruit shelf life assessment

To determine shelf life characteristics, fruits of similar size and developmental stage were harvested at commercial maturity. Several physical and chemical fruit quality attributes, such as pulling force (the required force required to detach the fruit from the receptacle), fruit weight, total soluble solids (TSS) and titratable acidity (TA) were evaluated daily through six days postharvest. TSS (%) of the raspberry juice was measured with a digital refractometer (PR-32 α Palette; Atago, Japan). For titratable acidity evaluation, 2 ml of fruit juice in 50 ml dH_2_O was titrated with 0.1 M NaOH to pH 8.2 and expressed as percentage of citric acid (%). To evaluate the retention force needed to remove the torus from the fruit, we adapted a fruit hardness tester (FHT200, Extech) with a 5 mm Penetrometer Tip. Fruit was attached to the instrument with a clamp and the force needed to remove its receptacle was recorded daily for twenty five fruits. Fruit weight was recorded daily for 25 fruits and the percent weight loss was calculated by taking the difference between initial weight and final weight divided by initial weight. After assessing physiological characteristics, all samples (20 fruit/replicate; three independent biological replicates) were frozen in liquid N_2_ and stored at −80 °C for further analysis.

### RNA extraction and qPCR assays

Total RNA extraction, DNase treatment and cDNA synthesis were performed as described previously.^38^ Quantitative reverse transcription PCR (qPCR) was conducted for 12 genes chosen to represent a variety of biological functions during the ripening and different developmental stages (Supplementary Information Supplementary Table S1). Gene-specific primers were designed using Primer Express (v3.0, Applied Biosystems, Carlsbad, CA, USA; Supplementary Information Supplementary Table S1). Two micrograms of total RNA were treated with DNaseI (Invitrogen, Burlington, ON, Canada) prior to cDNA synthesis using Superscript II reverse transcriptase (Invitrogen, Canada). PCR reactions were performed in 10 μl, containing SYBR Green master mix (0.2 mM dNTPs, 0.3 U Platinum Taq Polymerase (Invitrogen), 0.25× SYBR Green, and 0.1× ROX), 20 ng of cDNA and 300 nM of each primer. Four biological and three technical replicates for each reaction were analysed on a CFX96 Real-Time PCR Detection System (BioRad, Mississauga, ON, Canada) with a first step of 95 °C for 2 min followed by 40 cycles of 95 °C for 15 s and 60 °C for 1 min. Melting curves were generated using the following program: 95 °C for 15 s, 60 °C for 15 s, and 95 °C for 15 s. The expression of each gene was normalized to that of three reference genes (FvAct, His3 and GAPDH), and was quantified using the 2−ΔΔCt method.^39^ The results of the target genes relative transcript abundance are presented as a mean value of the three assay replicates compared with the mean of the three control values.

### Scanning electron microscope (SEM) analysis

Sample preparation was performed as described in Bray *et al.*^40^ Samples were fixed in 2.5% glutaraldehyde; 2% paraformaldehyde in 0.1 M phosphate buffer then washed three times in 0.1 M phosphate buffer for 10 min each. Ethanol dehydration series was conducted followed by three changes of 100% hexamethyldisilazane (HMDS) for 30-minute durations. After the third change, samples remained in HMDS until all of the solution evaporated. Samples were sputter-coated with carbon using a Nanotek SEMprep 2 sputter coater. Imaging and quantification analyses were conducted using a Zeiss EVO LS15 EP-SEM. The Zeiss EVO SEM with LaB6 electron source has a resolution of ~100 nm and equipped with a Bruker energy dispersive X-ray spectroscopy (EDS) system with a silicon drift detector with a resolution of 123 eV and a 10 mm^2^ window area.

### Phospholipase D activity assay

Phospholipase D Assay Kit was used following the manufacture’s recommended protocol (Sigma, Saint Louis, Missouri, USA, Cat. No.MAK137). In this assay, PLD hydrolyzes phosphatidylcholine to choline, which is determined using choline oxidase resulting in a colorimetric (570 nm) /fluorometric(*λ*_ex_=530/*λ*_em_=585 nm) product, proportional to the PLD activity in the sample. Three technical and three biological replicates were used in this assay, initial and final absorbance were measured at 570 nm using Epoch 2 Microplate (Biotek, Nepean, Ontario, Canada). The slope of the calibrator curve was used to calculate the phospholipase D activity of the sample using the following equation:

PLDActivity(units/L)=[A570(final)−A570(initial)]×n(slope×t)

where *t*=enzyme reaction time (20 min in standard assay) and *n*=dilution factor. Unit definition: one unit of PLD catalyzes the formation of 1 mmole choline per min under the assay conditions (pH 7.4).

### Statistical analysis

Data from the two consecutive growing seasons (2015 and 2016) were used in this analysis. Statistical analyses were performed in SAS v9.4 (SAS Institute, Cary, NC, USA). To meet assumptions of normality and heterogeneity of variance, data were log_10_ transformed prior to analysis using general linear models (PROC Mixed). Significant differences were determined by Tukey’s HSD test with overall *α* = 0.05.

## Results

### Physicochemical characteristics

The effect of hexanal on fruit quality, and physiological parameters that determine shelf life characteristics of raspberry fruits are provided in Table 1. As expected, TSS increased as the fruit approached maturity and throughout the storage period, both between treated and untreated fruits (Table 1). However, TA was relatively higher at harvest and declined progressively with the advance in storage duration. Statistical analysis did not show a significant effect of hexanal applications on the TA or the TSS. During the 6 days storage period, the amount of weight loss was significantly lower in the treated fruit after second and third day postharvest then returned to similar rate in both treated and control fruits for the remaining storage period (Table 1). Despite the minor effect of hexanal on weight loss of the fruit, the overall weight loss during the storage period was 30.2% in untreated fruits, compared to 25.9% in treated fruits, (Table 1).

**Figure 2:**
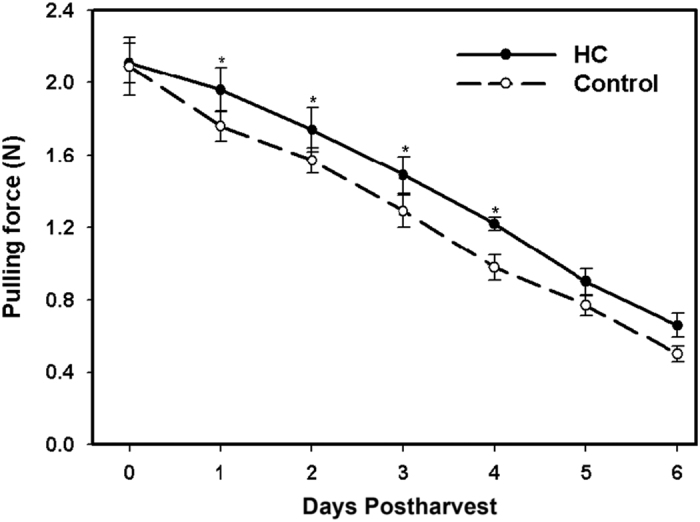
Tension force in control and hexanal treated (HC) raspberry fruits throughout six days post-harvest. Each value represents an average of twenty five fruit measurements of the tension force needed to remove the receptacles from their fruits. Asterisks indicate significant differences between control and hexanal treatment at the same storage time (*P*⩽0.05).

Fruit removal force, the pulling force required to remove a berry from its receptacle, was measured and used as an indicator of fruit softening. Hexanal application significantly increased the pulling force on the treated berry fruits compared to the control (Figure 2). All fruits showed continuous decline in the pulling force during subsequent storage at 20 °C, but the rate of decline was significantly slower in the treated fruits compared to control during the first 4 days of the storage period. During the first four days of postharvest, treated fruits required significantly higher amounts of force to remove the fruit from the receptacle (1.96–1.49 N) compared to the control (1.76–0.98 N), (Table 1). No significant difference was observed at the last two days postharvest. Under field conditions, it was very noticeable that the control fruits fell to the ground while the hexanal treated remained attached and still required minimal force to be separated from their receptacles (Supplementary Figure 1).

**Figure 3:**
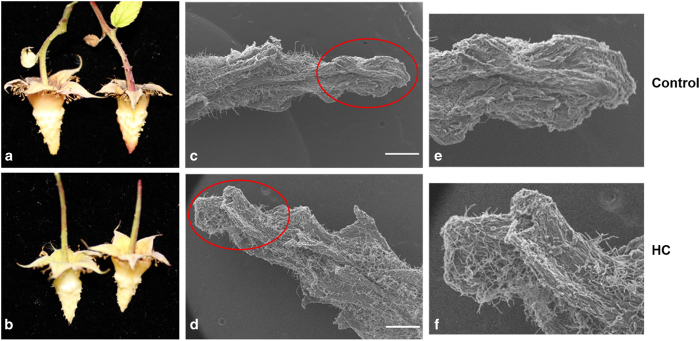
Histological observations of raspberry receptacles using SEM analysis. (**a**, **c** and **e**) represent control, while (**b**, **d** and **f**) represent hexanal treated fruits (HC). (**e** and **f**): a higher magnification of the receptacle tips showing an abundant epidermal hairs on the hexanal treated (**f**) while no epidermal hairs were observed on the receptacles of the control fruits (**e**). Scale bars are 500 μm.

Scanning electron microscope (SEM) analysis was conducted in order to better understand the mechanisms underlying the correlation between hexanal application and the enhancement of fruit retention. This analysis showed an abundant accumulation of epidermal hairs on the tip of the receptacles of the treated fruits while no epidermal hairs were observed on the tip of the untreated (Figure 3). Epidermal hairs were present in a similar quantity on the bases of both treated and control fruit receptacles.

### Hexanal altered gene expression

**Figure 4:**
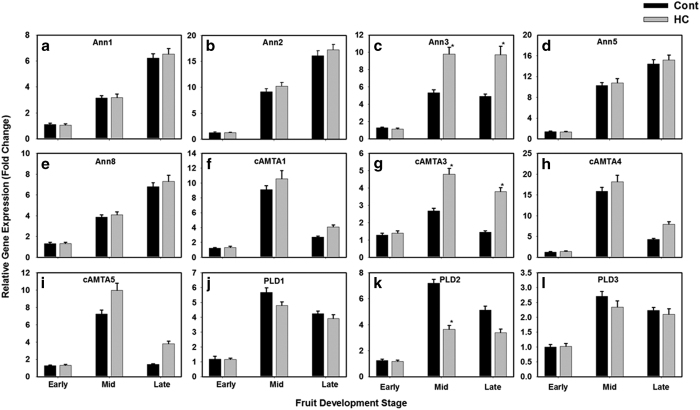
Transcript expression pattern measured by qRT-PCR of five annexins gene members (**a**–**e**), four calmodulin-binding transcription activators (**f**–**i**) and three phospholipase D genes (**j**–**l**) during fruit development of raspberry. Expression was determined relative to three housekeeping genes (His3, GPDH and Actin). Data represent the mean (±standard deviation, s.d.) of four biological replicates each with three technical replicates.. Statistically significant differences from Control (Cont) and hexanal treated (HC) at the same stage of fruit development are indicated by (*) for the probability levels (*P*<0.05).

The expression patterns of three phospholipase D genes, five annexin genes and four calmodulin-binding transcription activator were quantified during fruit developmental and ripening in response to hexanal application. The five members of annexin group showed a constant increase in their transcript levels throughout the fruit development stages. (Figures 4a–e). All annexin genes showed the same trend with a higher magnitude for Ann2 and Ann5. This pattern was common between treated and untreated fruits, however, hexanal significantly increased the transcript level of Ann3 at mid and late development stages (Figure 4c). Analysis of expression data of the four members of calmodulin-binding transcription activator showed that their transcript levels reached their peak at the mid stage of fruit development then declined (Figures 4f–i). Hexanal treated fruits did not show a different response, except a significant higher magnification for cAMTA3 at mid and late development stage and for cAMTA5 at late stage (Figures 4g and i).

The transcript levels of PLDs increased during fruit development, and attained their maximum transcript level at the mid-stage (Figures 4j–l). The highest level of expression was observed for PLDα1 and PLDα2, while PLDα3 had the lowest transcript level. From the PLDs, PLD2 was the only gene altered by hexanal as its transcript level showing a significant reduction at the mid stage of fruit development (Figure 4k). Nevertheless, the expression of the calmodulin-binding transcription factors and the phospholipase D genes showed a similar pattern during fruit development, the decline of the transcript level was more progressive in the PLD genes.

**Figure 5:**
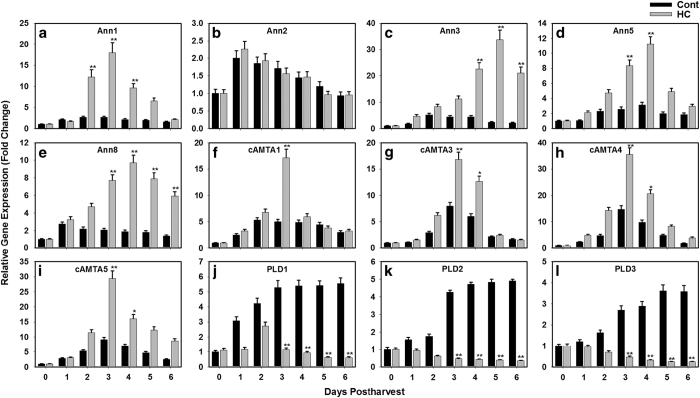
Hexanal applications altered the transcript levels of several genes involved in fruit ripening in raspberry. Transcripts were assessed by qRT-PCR of five annexins gene members (**a**–**e**), four calmodulin-binding transcription activators (**f**–**i**) and three phospholipase D genes (**j**–**l**) throughout six days postharvest. All fruit were stored at 20 °C until sampling.). Data represent the mean (±s.d.) of four biological replicates each with three technical replicates.. Expression data were normalized using three housekeeping genes (His3, GAPDH and Actin). Statistically significant differences from Control (Cont) and hexanal treated (HC) at the same day of postharvest are indicated by (*) and (**) for the probability levels (*P*<0.05) and (*P*<0.01), respectively.

A similar analysis of gene expression was conducted of these 12 genes throughout 6 days postharvest for hexanal treated and untreated fruits (Figures 5a–l). The statistical data analysis represented in this figure was performed to compare between hexanal treated and untreated samples at the same day of postharvest. The observation of the effect of hexanal on annexin gene expression revealed a progressive increase in their transcripts until the fourth day of postharvest before starting to decline (Figures 5a–d). The only exception of this trend in the annexins expression pattern was for Ann2 gene as its expression was the lowest and its level remained relatively constant throughout the postharvest period (Figure 5b). Interestingly, expression data for the four members of calmodulin-binding transcription activator showed a similar response to annexins transcripts due to hexanal applications. However, the progressive increase in the cAMTA gene transcripts reached the highest level at the third day postharvest then declined until day 6 (Figures 5f–i). Expression of PLDs in the untreated fruit showed a continuous increase in their transcript levels. In contrast, their transcript levels progressively declined during the six days of storage period due to hexanal treatment and only trace levels of transcript could be detected at the last four days (Figures 5j–l).

### Anatomical and structural observations in hexanal- treated fruits

**Figure 6:**
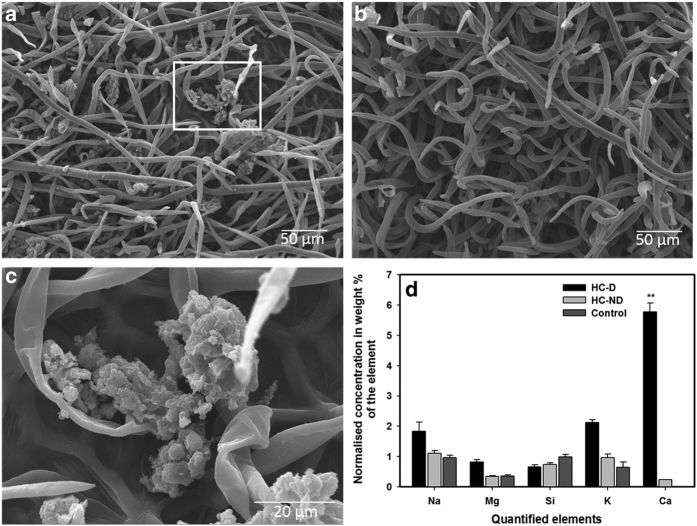
Structural observations of the drupelets of raspberry fruits by SEM-EDS analysis. Comparison of the epidermal hairs of the hexanal treated fruit drupelets (**a**) and control fruit (**b**) at their medium development stage. (**c**) shows a higher magnification view of the highly localized crystal deposition structures observed on the treated drupelet epidermal hairs. (**d**) showing data output generated from plots of the original spectrum of the EDS analysis. Each value represents an average of twelve samples. EDS analysis was conducted on non-treated fruit (Control), HC- (**d**): hexanal treated showing deposition structure, HC-ND: hexanal treated in an area with no crystal structure. Asterisks indicate significant differences in calcium level (*P*⩽0.01).

In order to strengthen our understanding of the effect of hexanal in altering fruit structure, photomicrographs, of the external (berry) and internal (receptacle) raspberry tissue samples were assessed during the different development stages using SEM. Hexanal applications at early or late stage of fruit development did not alter the external fruit structure (Supplementary Figure S2). However, fruits sprayed during the ideal, mid-stage of ripening showed a noticeable presence of crystalline deposition on the external surface of the drupelets (Figure 6a).

**Figure 7:**
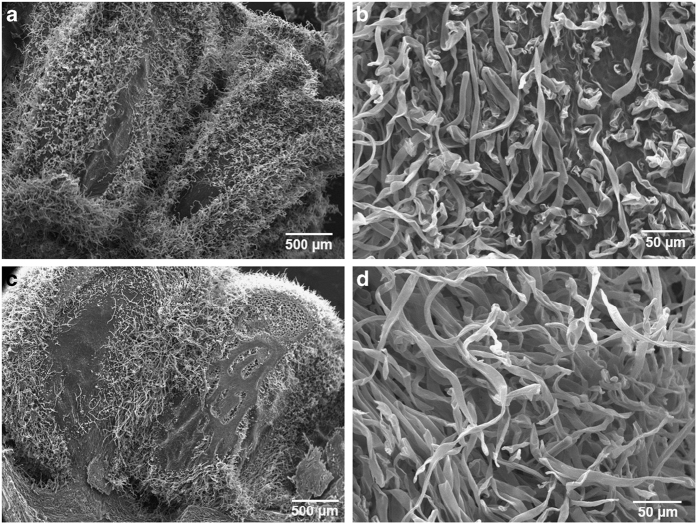
SEM analysis showing the internal structure of the raspberry fruit drupelets. (**a** and **b**): control fruit, (**c** and **d**): hexanal-treated fruit. Images were taken in the fruits at the medium developmental stage. No difference was observed in the internal structure of the fruit due to hexanal applications. The position of the seeds and the junctions between drupelets are shown in figures (**a** and **c**).

Energy Dispersive X-ray Spectrometer (EDS) analysis was conducted in order to identify the nature of these crystal depositions. This analytical technique allowed us to conduct elemental analysis and to compare between two areas on the same sample. Na, Mg, Si, K and Ca were quantified in the external drupelets of the control samples and in the areas with or without the crystalline depositions present in the hexanal-treated samples. When these elements were quantified, Ca was the most abundant and distinguishable element in the crystalline structures appearing on the drupelets of the treated fruits (Figures 6a and c). EDS analysis revealed that Ca represent an average of 6% of the total mass in these crystalline structures which was below the detectable level in other areas that were not showing any crystalline structure (Figure 6d). No significant difference was observed in the other quantified elements (Na, Mg, Si and K). The average total mass of these elements was 0.5–2% in all quantified areas of the treated and control tissues. In order to understand whether calcium accumulation can be observed in the internal fruit structure, longitudinal sections of the drupelets were examined using SEM. Unlike the case of the exocarp or the epidermal hairs, there were no visible signs of calcium deposits or structure differences in the mesocarp of treated and control fruits (Figures 7a–d). However, the filamentous epidermal hairs were more abundant in the treated fruits (Figure 7d).

In conclusion, the effect of hexanal on altering fruit structure was development stage-dependent and exclusive on the exocarp and epidermal hairs.

### Changes in PLD activity during fruit development and postharvest

To evaluate the potential role of hexanal in the inhibition of phospholipase D and the extension of shelf life, PLD activity was assayed at various stages of development in raspberry fruits. In this colorimetric assay, phosphatidylcholine is converted to choline by PLD, where choline is then oxidized by choline oxidase. The intensity of the colored reaction product is directly proportional to the activity of PLD in the sample.

**Figure 8:**
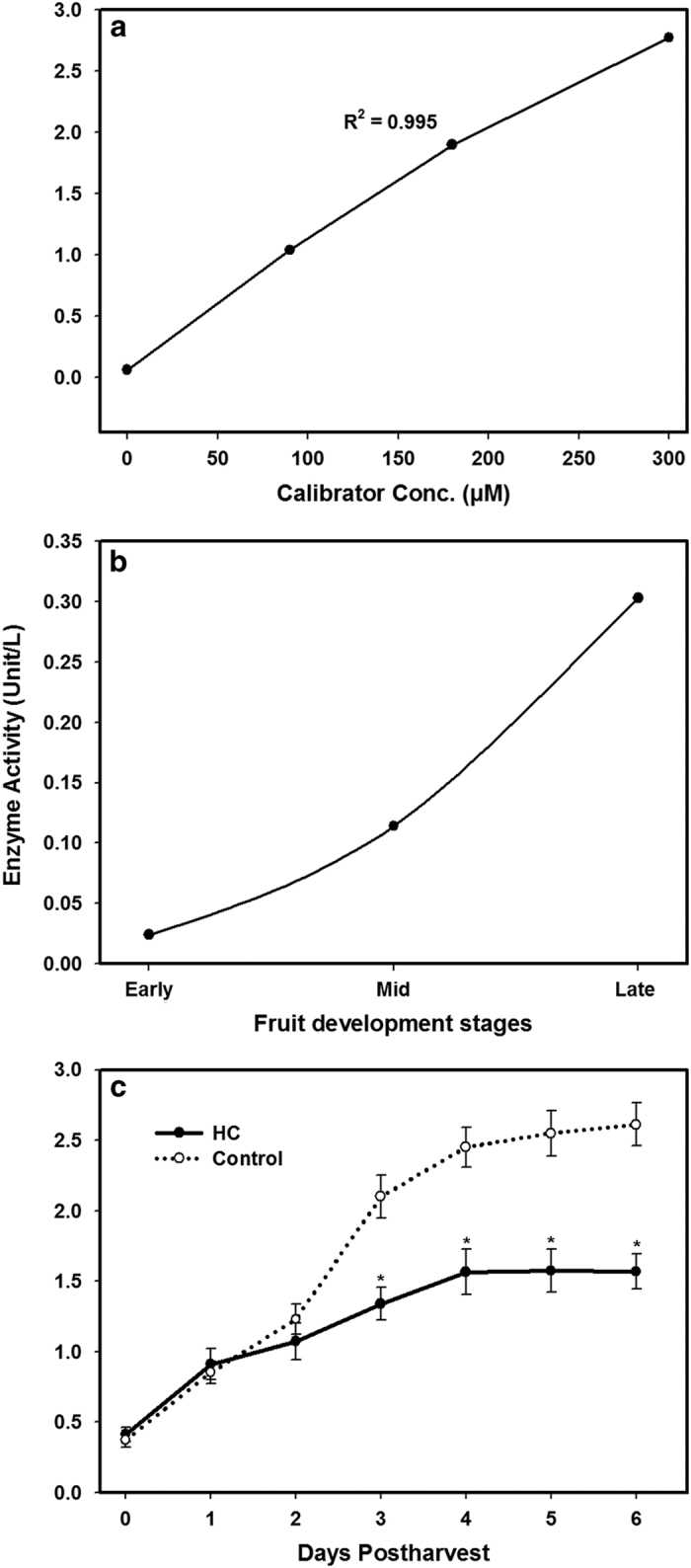
PLD activities observed in different stages of raspberry fruit development and during six days postharvest. (**a**) Quantification of the PLD activity in a series of different concentrations (0, 90, 180 and 300 μM) of the Calibrator (Choline); (**b**) shows the PLD activity at three different fruit developmental stages. (**c**) shows the PLD activity in control and hexanal treated (HC) fruits throughout the 6 days postharvest. PLD activity was measured in the total homogenates of control and hexanal-treated fruit. The hexanal treatment caused a significant reduction in the rate of increase of PLD activity after the third day postharvest. In figure (**c**), statistically significant differences from Control and hexanal treated (HC) at the same day of postharvest are indicated by (*) for the probability levels (*P*<0.05).

One unit of PLD catalyzes the formation of 1 mmol choline per minute under the assay conditions (pH 7.4). PLD activity was analyzed by monitoring the liberation of choline by unit/l (Figures 8a–c). The early stage of fruit development contained very little PLD activity (0.024 unit per L). However, PLD activity increased during fruit development and reached a maximum level of 300 μM choline (0.3 unit per L) released at the late stage of fruit development (Figure 8b). These results suggested that PLD activity increased during fruit development and ripening and play a key role in fruit softening as observed earlier. Moreover, the PLD activity assay was also conducted throughout the 6 days of postharvest in both hexanal-treated and -untreated fruits This assay showed a continuous increase in the PLD activity during storage in all fruits; however, the rate of increase was significantly slower in the hexanal-treated fruit during the last four days of the postharvest period (Figure 8c). For instance, during the first three days of postharvest, the amount of released choline increased from 0.37 (unit per L) to 1.23 (unit per L) in control fruit and from 0.41 (unit/l) to 1.07 (unit/l) in hexanal-treated fruit. At day three, this value reached 2.1 (unit per L) and it continued to increase until it attained a value of 2.61 (unit per L) at day six in the control fruit (Figure 8c). By contrast, hexanal applications reduced this rate of increase of PLD activity from 1.34 (unit per L) at day three to reach a maximum value of 1.56 (unit per L) at the end of the storage period. Taken together, our data revealed that hexanal can reduce the increased rate of the PLD activity by 12 % after a week postharvest.

## Discussion

The control of fruit softening is an important target for the improvement of fruit quality as fruit firmness appears to affect susceptibility to fungal pathogens.^41^ This is particularly true for raspberry, which is very tender by nature and has an extremely short shelf life. Biochemical and genetic studies of fruit softening indicated that cell-wall processing is the result of coordinated expression of several families of genes encoding cell-wall metabolism related proteins.^7–11^ However, many efforts to suppress expression of cell wall-degrading enzymes have not provided the insight needed to genetically engineer fruits whose softening can be adequately controlled.^12,13^ Phospholipase D (PLD), is the key enzyme involved in initiating a cascade of catabolic events that leads to the eventual deterioration of the membrane phospholipids.^2,3^ In this study, we tested the hypothesis that hexanal application could eventually suppress the PLD activity and thus lead to enhanced membrane stability, and thereby increased longevity of raspberry fruit. To achieve this end, we have used multidisciplinary approaches including gene expression, physiological and histological characterization and enzymatic activity in order to strengthen the understanding the effect of hexanal in enhancing raspberry fruit shelf life. Since the action of hexanal is so specific, there was little effect on the TSS and TA, while it had a striking effect on fruit retention. This is in in agreement with Anusuya *et al.*^42^ who indicated that pre-harvest spray of hexanal assisted in retention of mango fruits in the orchards for about 2–3 weeks. Extending the fruit retention time will significantly reduce the postharvest losses, resulting in a significant economic benefit. Moreover, this might be used as an indicator of enhanced fruit firmness especially since the growth regulators that target fruit retention may also be used to improve firmness, as both abscission and ripening are ethylene-mediated events in fruit development.^43^ In raspberry fruit there is some controversy as to whether the fruit are truly climacteric. Although there is a large increase in the rate of ethylene production, there is no accompanying burst in the rate of respiration.^44^ However, there are many features of fruit ripening that are common to both climacteric and non-climacteric species indicating that there is overlap in the molecular mechanisms underlying the ripening process in both types, despite the differences in respiration and ethylene production.

Part of the explanation of this increase in fruit retention was revealed by SEM analysis showing an abundant amount of the epidermal hairs on the tips of the treated fruit receptacles enhancing the attachment between fruits and their receptacles, thus increasing force required to remove the fruit receptacle.

Moreover, histological observations by SEM and EDS confirmed that there is calcium deposition on the epidermis of the drupelets of the hexanal treated fruit. Calcium has received considerable attention in recent years because of its desirable effects in controlling fruit ripening and quality by delaying ripening and maintaining firmness. For instance, increasing the calcium concentration of fruit through pre-harvest sprays and postharvest calcium treatments maintains firmness and prevents decay in both climacteric and non-climacteric fruits.^45,46^ Calcium is recognized also as a critical element for rigidifying cell walls by cross-linking with pectins.^21,47^ However, to our knowledge, this is the first report suggesting a potential crosstalk between hexanal application and calcium accumulation. Gene expression data also supports this hypothesis as hexanal treatment increased the transcript levels of four annexins and three calmodulin-binding transcription activators during raspberry postharvest storage. Previous studies suggested the potential roles of annexins in fruit development and ripening. For instance, there was a positive correlation between the stage of fruit development in strawberry and transcript levels of two annexins genes.^48^ Data analysis from a proteome survey of strawberry fruits during ripening suggested a role of annexins in fruit ripening,^49^ however, an independent regulation of individual annexin was suggested probably due to the large protein family with multi-functions.

Calcium-sensing proteins called calmodulins have been found to regulate both enlargement and ripening of tomatoes.^50,51^ One of the important findings in the study of Yang *et al.*^51^ was that the suppression of calmodulin genes could be critical for initiating ethylene-independent ripening process. In this study, the expression of three calmodulin-binding transcription activators (cAMTA1, cAMTA4 and cAMTA5) was differentially altered in response to hexanal application which reinforces the clues for a potential effect of hexanal on calcium binding proteins that function as signal sensors.

Our studies also demonstrate a clear correlation between PLD activity, and PLDs gene expression at various stages of raspberry fruit development as well as during the storage period. A similar analysis was conducted using normal and antisense PLD in ‘Celebrity’ tomatoes.^1^ These results conclusively showed that PLD expression was reduced during fruit ripening by the introduction of antisense PLD alpha cDNA in ‘Celebrity’ tomatoes. Hexanal treatment caused a clear PLD activity inhibition in raspberry fruits in a similar way that the antisense PLD tomato lines showed a reduced level of PLD activity. In the same report, microsomal PLD activity in strawberry fruit membranes was found to be stimulated by calcium. These results, together with our show a clear correlation between hexanal application, cellular calcium stimulation and PLD activity inhibition. However, the complete sequence of signal transduction events involved in this link requires further investigation.

Despite the fact that raspberries appear to be a very responsive system to hexanal application, the stage of application of pre-harvest hexanal treatment seems to be very important in order to modulate the PLD inhibition and to achieve optimum effect. For instance, the histological observations reveal that the calcium depositions will occur exclusively if the fruits are treated in their mid-development stage. Misran *et al.*^39^ also indicated that the stage of fruit application is crucial since PLD inhibition has very little effect once the membrane starts to deteriorate and lipid catabolic cascades are initiated. For example, tomato fruits are treated at the mature green stage to obtain the best results in terms of colour and flavour development. Whereas berry fruits such as cherry should be treated about 15 days before harvest to maximize their shelf life to 30 days at 4 ° C.^5^

In conclusion, this work clearly demonstrated the important role hexanal can play in reducing PLD transcript levels and activity as well as altering some of the calcium binding proteins that function as signal sensors. Thus, hexanal application might be a desirable and suitable technology to enhance the postharvest shelf life of raspberry in light of its extremely perishable nature. Hexanal, in spite of its chemically inclined name, is a natural compound produced by all plants as a volatile defense mechanism. It is also a GRAS (generally regarded as safe) compound approved by the FDA as a food adjunct.^52^ These two characteristics should alleviate any fears consumers might have on its use on edible produce such as raspberry. It would be also interesting to determine if the calcium accumulation due to hexanal application will affect the edible quality and taste of fruits. Further studies could be used to develop an understanding of the biochemical mechanism of interaction between hexanal and the intercellular calcium in the overall regulation of PLD in extending fruit shelf life.

## Figures and Tables

**Table 1:** Quality parameter of raspberry fruit after pre-harvest hexanal (HC) applications

*Parameter*	*Treat*	*Storage time (d)*						
		*0*	*1*	*2*	*3*	*4*	*5*	*6*
Pulling force (N)	Ctrl.	2.09 (0.18)	1.76 (0.15)	1.51 (0.12)	1.29 (0.11)	0.98 (0.08)	0.77 (0.06)	0.50 (0.04)
	HC	2.11 (0.19)	1.96* (0.13)	1.74* (0.13)	1.49* (0.10)	1.22* (0.09)	0.85 (0.07)	0.66 (0.05)
TSS (°Brix)	Ctrl.	10.21 (0.72)	10.12 (0.61)	10.27 (0.48)	1063 (0.64)	10.71 (0.80)	10.81 (0.61)	10.86 (0.71)
	HC	10.13 (0.64)	9.94 (0.71)	10.14 (0.52)	10.32 (0.70)	10.74 (0.71)	10.84 (0.64)	10.82 (0.68)
TA (g/100 ml citric acid)	Ctrl.	1.91 (0.08)	1.84 (0.09)	1.73 (0.11)	1.71 (0.10)	1.28 (0.07)	1.24 (0.06)	1.10 (0.10)
	HC	1.91 (0.05)	1.90 (0.07)	1.83 (0.06)	1.77 (0.08)	1.34 (0.06)	1.16 (0.07)	1.11 (0.11)
Weight (g)	Ctrl.	25.84 (1.78)	23.93 (2.22)	21.52 (1.84)	20.61 (1.94)	20.23 (2.10)	19.94 (2.45)	18.04 (2.31)
	HC	26.22 (2.41)	25.81 (2.13)	24.64* (1.79)	23.83* (2.04)	21.81 (2.11)	20.61 (2.56)	19.42 (1.87)

Values represent the mean of four biological replicates. Means followed by asterisks indicates significant differences between control and hexanal treatment at the same storage time (*P*⩽0.05). Standard deviations are shown between brackets.
