# Supplementary material for: Phospholipase D inhibition by hexanal is associated with calcium signal transduction events in raspberry
Source: Hortic Res. 2017 Sep 13;4:17042–. doi: 10.1038/hortres.2017.42 (PMC5596117; doi:10.1038/hortres.2017.42)
Supplement: Supplementary Table S1 [file hortres201742-s1.docx]

| **primer name** | **Sequence** | **Accession no.** |
| --- | --- | --- |
| q-FaCAMTA1-Fw | CTTGAGGCTGAGAATGGTGATAG | XM_011466647.1 |
| q-FacAMTA1-Rev | AGTTGTTCCTGGGAAAGAGAAG |  |
| q-FacAMTA3-Fw | CCTACTGGGAGACTGTTGAAAG | XM_004288145.2 |
| q-FacAMTA3-Rev | AGCTGGTCATGAGAAAGAGAAG |  |
| q-FaCAMTA4-Fw | GAATCTGGTAGGAGTGCAGAAG | XM_011459191.1 |
| q-FaCAMTA4-Rev | CTCAGACTCGACAGTGTCTCTA |  |
| q-FaCAMTA5-Fw | TACCCTCAAAGAAGCCAAGAAG | XM_004295055.2 |
| q-FaCAMTA5-Rev | TGCCACTGGTAAAGGAGTATTG |  |
| q-FvAnn1-Fw | ATGCCGAAACTTATGGAGAGG | XM_004299596.2 |
| q-FvAnn1-Rev | AGCAGGATCAAGAGTCCAAAG |  |
| q-FvAnn2-Fw | GAGAGCAACTGCCAAACTATTG | XM_004288159.2 |
| q-FvAnn2-Rev | CTAGTAAGGGCACCTTCATCTG |  |
| q-FvAnn3-Fw | CCTCCTGAAAGGGATGCTAAA | XM_004307086.2 |
| q-FvAnn3-Rev | CCATCAAGTGGTGAGGAGATG |  |
| q-FvAnn5-Fw | GTATCGAGGGCTGAGATTGATT | XM_004299614.2 |
| q-FvAnn5-Rev | CCCTGTAATTCCCAGAAGTCTC |  |
| q-FvAnn8-Fw | CTACAAGCACTCTGTGGAAGAA | XM_004307084.2 |
| q-FvAnn8-Rev | CATTGATCTCGTGACCGTCATA |  |
| q-Rasp-Actin-Fw | GGAGCACCCAGTTCTTCTTAC | GQ339772.1 |
| q-Rasp-Actin-Rev | GGCCTGGATAGCAACATACAT |  |
| q-Rasp-His3-Fw | CTCCGTGAAATCCGCAAGTA | AF304365.1 |
| q-Rasp-His3-Rev | AACTGCATGGCTCTGGAAA |  |
| q-Rasp-GAPDH-Fw | GCGGCTTCCTATGAACAAATC | KM353458.1 |
| q-Rasp-GAPDH-Rev | CCTCCCTGTGAAGAGAGAATTAG |  |
| q-Rasp-PLD1-Fw | GTGGGTATTGGCAAAGGAATTAG | AY758359.1 |
| q-Rasp-PLD1-Rev | CACTTGGGATTAGAGGGTTCTT |  |
| q-PLD2-Fw | TGACTCTGGGAGAGCTTCTTA | XP_004287315.1 |
| q-PLD2-Rev | CCCATCAAGCCATCTCTCTTT |  |
| q-PLD3-Fw | GTGGACAGATGGGTTGAGATAC | XP_011459180.1 |
| q-PLD3-Rev | CCTGAGACCAATGAGCATCTT |  |

Table S1 : Genes and gene-specific primers used for qRT-PCR
