# Supplementary figures and images for: Phospholipase D inhibition by hexanal is associated with calcium signal transduction events in raspberry
Source: Hortic Res. 2017 Sep 13;4:17042–. doi: 10.1038/hortres.2017.42 (PMC5596117; doi:10.1038/hortres.2017.42)

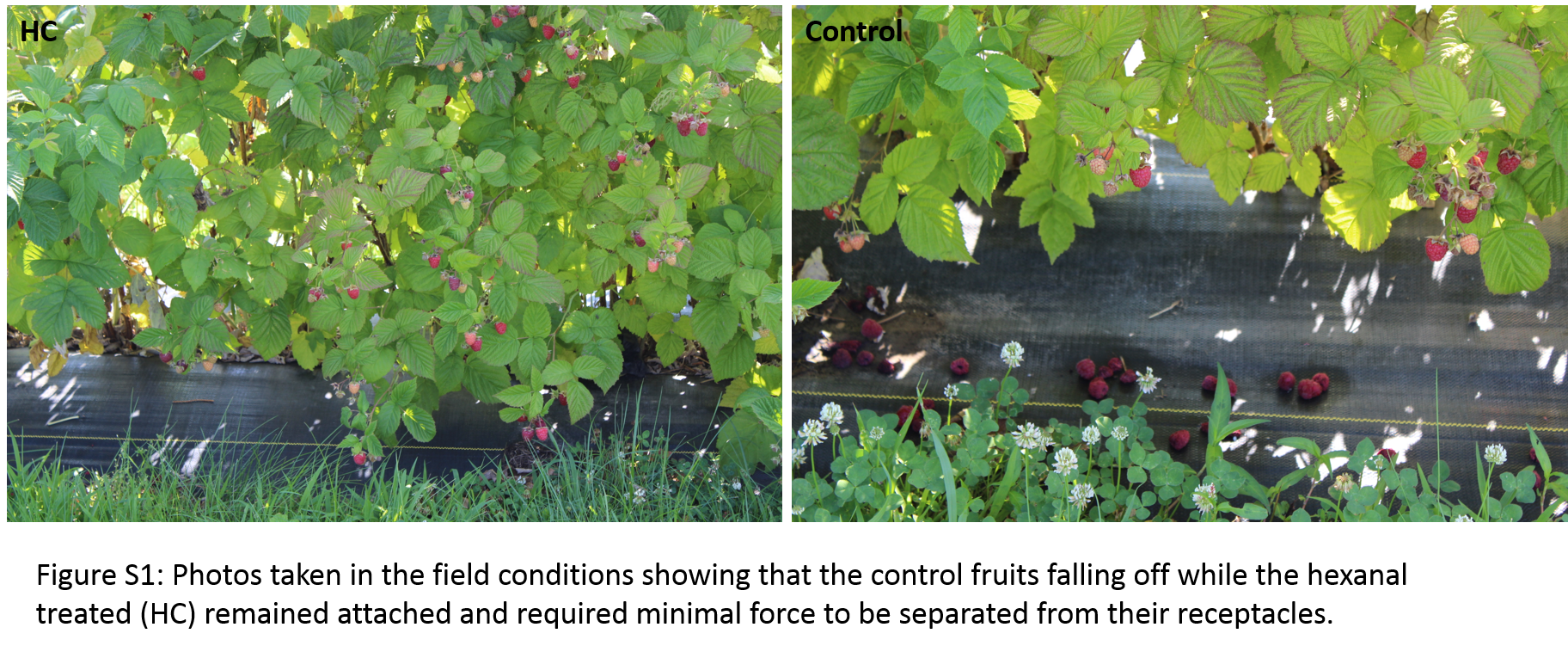

Supplement: Supplementary Figure S1 [file hortres201742-s2.tiff]

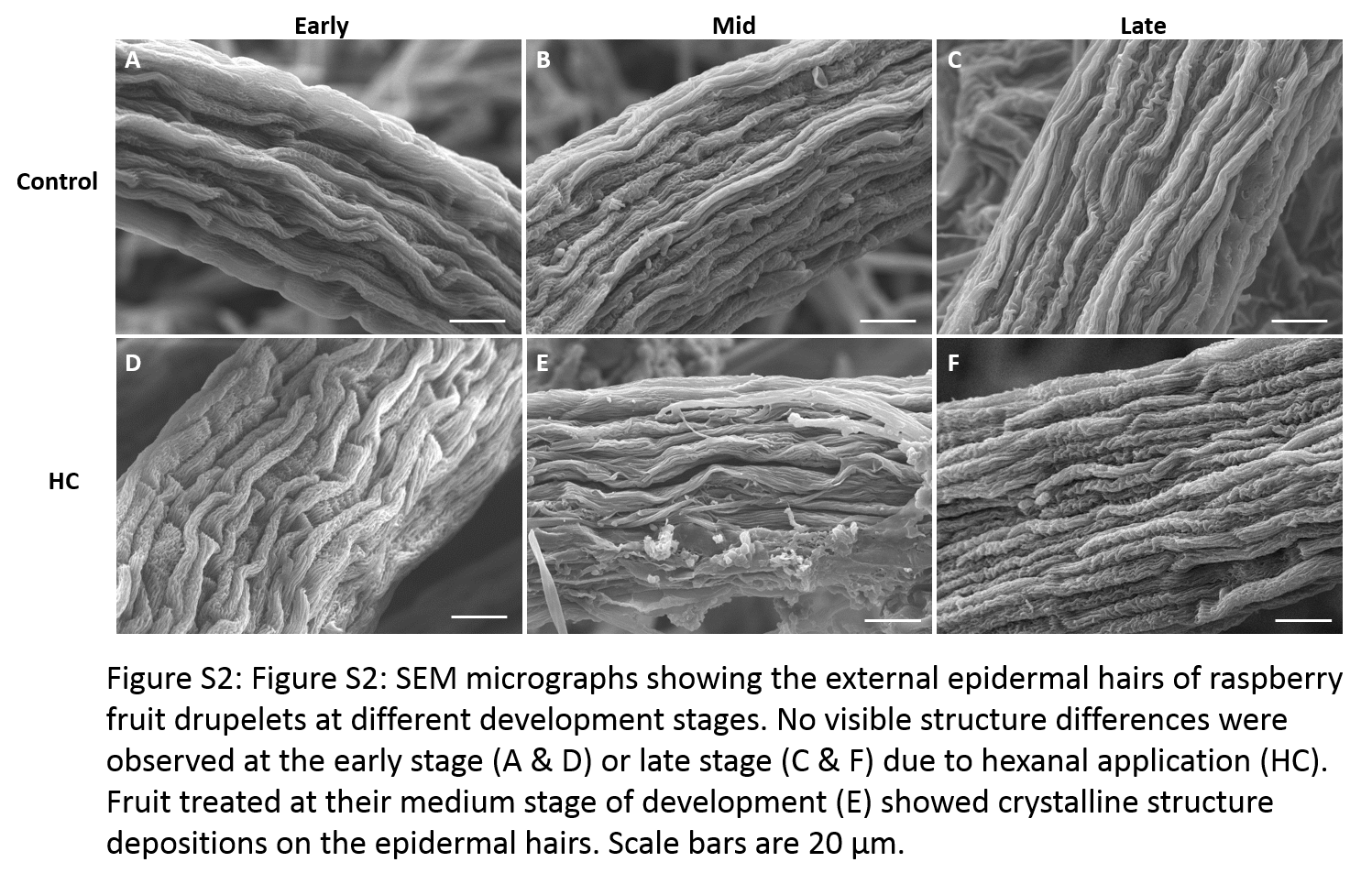

Supplement: Supplementary Figure S2 [file hortres201742-s3.tiff]
