# Supplementary material for: Phospholipase D inhibition by hexanal is associated with calcium signal transduction events in raspberry
Source: Hortic Res. 2017 Sep 13;4:17042–. doi: 10.1038/hortres.2017.42 (PMC5596117; doi:10.1038/hortres.2017.42)
Supplement: Supplementary Figure S3 [file hortres201742-s4.pdf]

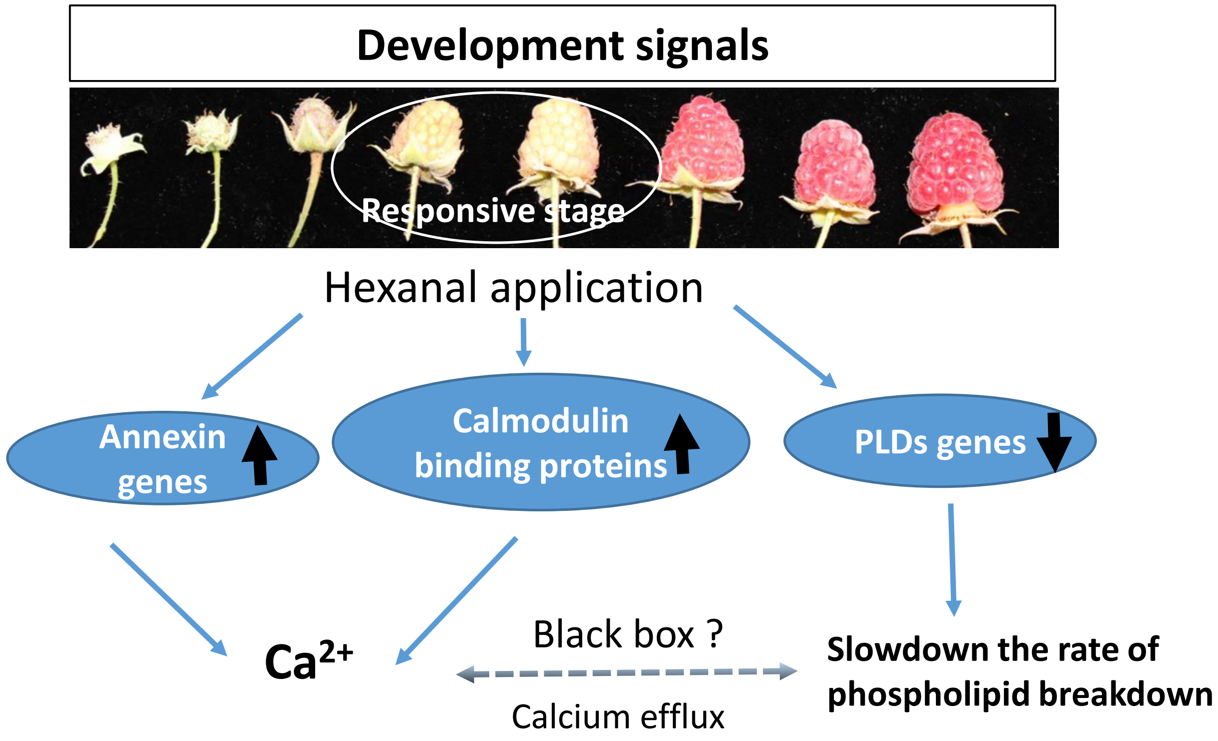

Figure S3: A proposed model showing the potential cross-talk between hexanal, PLD activity and calcium accumulation. The transcript levels of PLD genes were decreased by hexanal application while calmodulin binding proteins and annexin genes showed higher transcript levels. The potential role of hexanal in suppressing the PLD activity and stimulating the accumulation of cellular calcium requires more investigations.
